# Research on the Synergistic Effect of Total Ionization and Displacement Dose in GaN HEMT Using Neutron and Gamma-Ray Irradiation

**DOI:** 10.3390/nano12132126

**Published:** 2022-06-21

**Authors:** Rui Chen, Yanan Liang, Jianwei Han, Qihong Lu, Qian Chen, Ziyu Wang, Hao Wang, Xuan Wang, Runjie Yuan

**Affiliations:** 1 National Space Science Center, Chinese Academy of Sciences, Beijing 100190, China; liangyanan@nssc.ac.cn (Y.L.); hanjw@nssc.ac.cn (J.H.); chenqian16@mails.ucas.ac.cn (Q.C.); wangziyu211@mails.ucas.ac.cn (Z.W.); wanghao21c@mails.ucas.ac.cn (H.W.); wangxuan171@mails.ucas.ac.cn (X.W.); yuanrunjie20@mails.ucas.ac.cn (R.Y.); 2 Institute of Astronomy and Space, University of Chinese Academy of Sciences, Beijing 101408, China; 3 School of Physical Science and Technology, Yangzhou University, Yangzhou 225000, China; yzlqhaimenghuan@163.com

**Keywords:** displacement damage effect, total ionizing dose effect, gamma-ray, neutron, gallium nitride high electron mobility transistor

## Abstract

This paper studies the synergistic effect of total ionizing dose (TID) and displacement damage dose (DDD) in enhancement-mode GaN high electron mobility transistor (HEMT) based on the p-GaN gate and cascode structure using neutron and ^60^Co gamma-ray irradiation. The results show that when the accumulated gamma-ray doses are up to 800k rad(Si), the leakage-current degradations of the two types of GaN HEMTs with 14 MeV neutron irradiation of 1.3 × 10^12^ n/cm^2^ and 3 × 10^12^ n/cm^2^ exhibit a lower degradation than the sum of the two separated effects. However, the threshold voltage shifts of the cascode structure GaN HEMT show a higher degradation when exposed to both TID and DDD effects. Moreover, the failure mechanisms of the synergistic effect in GaN HEMT are investigated using the scanning electron microscopy technique. It is shown that for the p-GaNHEMT, the increase in channel resistance and the degradation of two-dimensional electron gas mobility caused by neutron irradiation suppresses the increase in the TID leakage current. For the cascode structure HEMT, the neutron radiation-generated defects in the oxide layer of the metal–oxide–semiconductor field-effect transistor might capture holes induced by gamma-ray irradiation, resulting in a further increase in the number of trapped charges in the oxide layer.

## 1. Introduction

As the representative of the wide bandgap semiconductor devices, the gallium nitride high-electron-mobility transistor (GaN HEMT) has excellent electrical performance, high-temperature resistance, high power, and resistance to extreme radiation environments, which could meet the needs of new-generation spacecraft energy systems [1,2,3,4]. When a nuclear-powered spacecraft works in space, in addition to radiation damage caused by energetic particles, the comprehensive radiation environment with neutrons and gamma rays could also lead to performance degradation or even device failure of electronic systems by displacement damage effects (DDD) and total dose effects (TID) [5,6,7,8,9]. According to the reports [10,11], at a distance of 5.3 m from the 461 MW nuclear reactor core, normalized by the power, the neutron flux with an energy greater than 3 MeV is 2.13 × 10^8^ n·cm^−2^·s^−1^·MW and an energy smaller than 0.4 MeV is 9.84 × 10^8^ n·cm^−2^·s^−1^·MW. Meanwhile, the gamma-ray dose rate is 0.08 Sv·s^−1^·MW. At present, the TID and DDD effects of GaN HEMT have been, respectively, studied using neutron and gamma-ray radiation. For the TID case, it involves the radiation characterization, failure mechanism, and mitigation design, while most DDD studies only focus on the radiation appearances under neutron and proton experiments. Moreover, some previous works show that the TID effect could be influenced by neutron radiation in bipolar transistors [8]. However, it is a pity that the synergistic effect of DDD and TID in GaN HEMT has not been reported so far. This is a challenge to evaluate the reliability of the electronic system in a comprehensive radiation environment involving neutrons and gamma-rays.

In this paper, the DDD and TID irradiation experiments are carried out with the 14 MeV neutron high-voltage multiplier and ^60^Co gamma-ray successively. The experiment devices are the enhancement mode GaN HEMT based on the p-GaN gate and the cascode structure. The characteristics of the TID effect under neutron irradiation are studied and the failure mechanisms of the synergistic effect of DDD and TID in the GaN HEMT are revealed.

## 2. Experimental Setup

**Figure 1:**
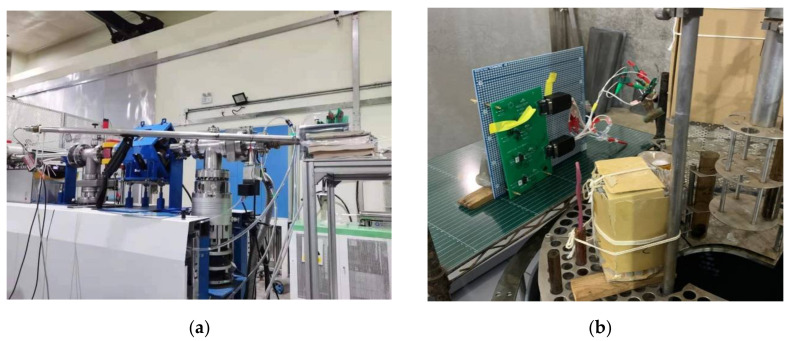
The experimental setup of (**a**) DDD and (**b**) TID test.

The DDD experiments of the GaN HEMT based on the p-GaN gate and the cascode structure were carried out by the 14 MeV neutron high-voltage multiplier at the China Institute of Atomic Energy. For p-GaN gate, GaN HEMT radiation, the neutron flux, and fluence were 5 × 10^7^ n/cm^2^/s and 3 × 10^12^ n/cm^2^, respectively. For the cascode structure GaN HEMT case, a flux of 2.6 × 10^7^ n/cm^2^/s and fluence of 1.3 × 10^12^ n/cm^2^ were adopted. The TID experiments of the GaN HEMT based on the p-GaN gate and the cascode structure were carried out using the ^60^Co gamma-ray source at Peking University, China. The dose rate was 100 rad(Si)/s in the experiments. The TID characteristics of the GaN HEMT were tested online at 50k rad(Si), 200k rad(Si), 500k rad(Si), and 800k rad(Si), respectively. Then, 168-hour annealing at room temperature was performed after total dose irradiation. The experimental setup of the DDD and TID test is shown in Figure 1.

**Figure 2:**
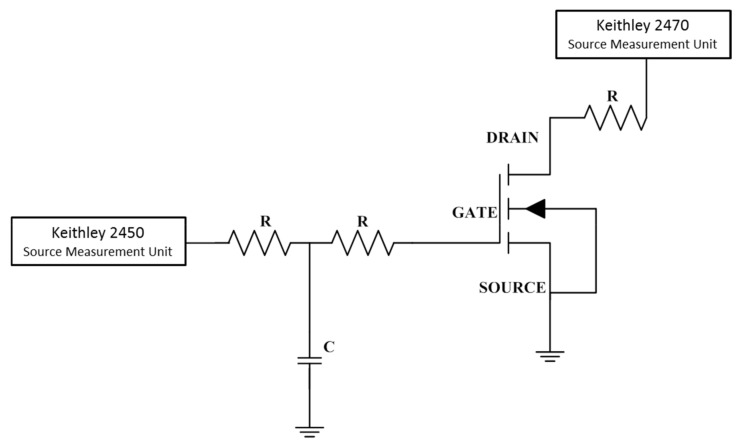
The schematic diagram of the DDD and TID test circuit.

The commercially available E-mode GaN HEMTs with p-GaN gate and cascode structure was adopted in the DDD and TID testing. The device parameters are shown in Table 1. As shown in Figure 2, the test circuit refers to the MIL-STD-750D. Keithley 2470 and 2450 source measurement unit (SMU) are connected to the drain-source and gate-source terminal of the device through resistance and capacitance, respectively, which provide drain-source voltage (V_ds_) and gate-source voltage (V_gs_). Additionally, the current-voltage and transfer curve characterization were also obtained by them. The GaN HEMTs were biased on off-state (V_ds_ = V_gs_ = 0 V) and on-state (V_ds_ = 1 V, V_gs_ = 2.6 V) during irradiation, respectively.

## 3. Experimental Results

### 3.1. Neutron Irradiation

**Figure 3:**
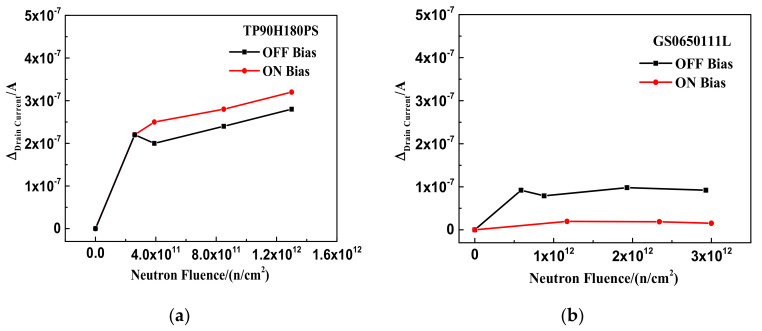
The relationship between the leakage current variations of the device and the neutron irradiation under the on-state and off-state: (**a**) TP90H180PS and (**b**) GS0650111L.

The relationships between leakage current variation and neutron irradiation fluence of TP90H180PS and GS0650111L are shown in Figure 3a,b. The leakage currents of TP90H180PS and GS0650111L before irradiation are 1.6 μA and 200 nA, respectively. When the neutron irradiation fluence accumulates to 1.3 × 10^12^ n/cm^2^ and 3 × 10^12^ n/cm^2^, respectively, the leakage current of the two-type devices increases slightly with neutron irradiation. The leakage current variations of the two-type devices under on-state radiation are 3.2 × 10^−7^ A and 1.5 × 10^−8^ A and under off-state radiation are 2.8 × 10^−7^A and 9 × 10^−8^ A, respectively.

**Figure 4:**
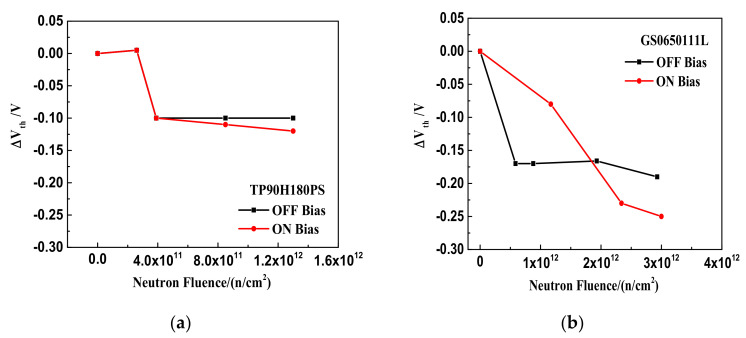
The relationship between the threshold voltage shifts of the device and the neutron irradiation under the on-state and off-state: (**a**) TP90H180PS and (**b**) GS0650111L.

Figure 4 shows the threshold voltage shifts as the function of neutron irradiation influence for the GaN HEMT based on the p-GaN gate and the cascode structure. It is shown that in Figure 4a the threshold voltage offsets of the TP90H180PS negatively shift about 0.1 V under on- and off-bias with neutron fluence of 1.3 × 10^12^ n/cm^2^. In addition, as shown in Figure 4b, the threshold voltage offsets of the GS0650111L also negatively shift under on- and off-bias with neutron fluence of 3 × 10^12^ n/cm^2^. The maximum threshold voltage shifts are approximately −0.25 V under on-bias and −0.2 V under off-bias, respectively.

### 3.2. ^60^Co Gamma-Ray Irradiation

**Figure 5:**
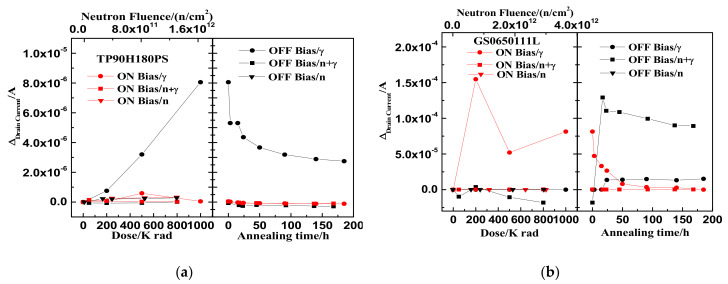
The variations of drain current with the neutron and gamma-ray irradiation and 168 h annealing under the on-state and off-state: (**a**) TP90H180PS and (**b**) GS0650111L.

After neutron irradiation, the TID effect of the GaN HEMT based on the p-GaN gate and the cascode structure were studied under the same bias condition using gamma-ray irradiation for a week. The electrical characteristics of the devices were tested before gamma irradiation. The pre-irradiation leakage currents were about 1.9 μA for TP90H180PS and 300 nA for GS0650111L under the off state, respectively, which were mostly consistent with those after neutron irradiation. It indicated that no annealing effect occurred in a week. Two types of devices without neutron irradiation were gamma-ray irradiated to study the influence of neutron irradiation on the TID effect. The fluences of neutron radiation are 1.3 × 10^12^ n/cm^2^ for TP90H180PS and 3 × 10^12^ n/cm^2^ for GS0650111L, respectively. The leakage current variations as the function of the accumulated TID dose and the DDD fluence are shown in Figure 5. The devices are biased on off-state (V_ds_ = V_gs_ = 0 V) and on-state (V_ds_ = 1 V, V_gs_ = 2.6 V) during irradiation, respectively. Figure 5a shows that when accumulated gamma-ray doses are up to 800k rad(Si), the leakage current of TP90H180PS biased on on-state is little changed under the combined effect of neutron and gamma-ray irradiation. However, the leakage current variation of TP90H180PS at off-state bias is 10 nA under the combined effect of the neutron and gamma-ray, which is less than 8 μA under the gamma-ray radiation and 280 nA under the neutron radiation. After 168 h of annealing at room temperature, the leakage current of the device mostly recovers to the normal value. As is shown in Figure 5b, exposed to accumulated gamma-ray doses to 800k rad(Si), the leakage current of GS0650111L at off-state bias is also much the same under the combined effect of neutron and gamma-ray irradiation. In constant, the leakage current of GS0650111L at on-state bias increases by 75 μA under the gamma-ray radiation at accumulated doses of 1M rad(Si). After annealing for 50 h at the on-state bias under gamma-ray irradiation, the leakage current mostly recovered to the normal value. However, at off-state bias the leakage current increases by 15 μA under the gamma-ray irradiation, 90 nA under the neutron radiation, and 100 μA under the combined effect of neutron and gamma-ray irradiation. While the device’s performance test passes after the TID annealing.

**Figure 6:**
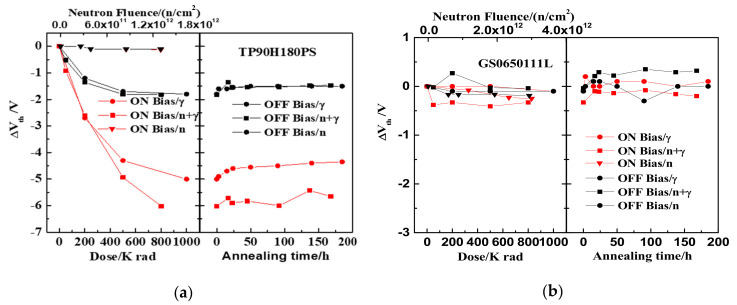
The variations of threshold voltage with the neutron and gamma-ray irradiation and 168 h annealing under the on-state and off-state: (**a**) TP90H180PS and (**b**) GS0650111L.

Figure 6 shows the threshold voltage shifts of TP90H180PS and GS0650111L as the function of accumulated TID dose and the neutron irradiation fluence. As shown in Figure 6a, when gamma-ray doses accumulate to 800k rad(Si), the threshold voltage of TP90H180PS at on-state bias negatively shifted about 6 V under the combined effect of neutron and gamma-ray irradiation, about 4.5 V under the gamma-ray irradiation and about 0.1 V under the neutron irradiation. While the threshold voltage of TP90H180PS at off-state bias both shifted about −1.7 V, under the combined effect of neutron and gamma-ray irradiation and the gamma-ray irradiation. After annealing at room temperature for 168 h, the threshold voltage of the device is still negatively shifted under the on and off state. The device’s function is abnormal after TID annealing. As shown in Figure 6b, when gamma-ray doses accumulate to 800k rad(Si), the threshold voltage of GS0650111L is little changed. In addition, under the combined effect of neutron and gamma-ray irradiation, the threshold voltage of GS0650111L biased on on-state shows a negative shift of about 0.3 V. After annealing at room temperature for 168 h, the threshold voltage returned to normal value and the device’s functional test passed.

## 4. Discussion

Figure 3 and Figure 4 show that the leakage current and the threshold voltage of the two-type GaN HEMTs exhibit slight degradation under neutron irradiation. It indicates that the two-type GaN HEMTs are not very sensitive to the DDD effect when the accumulated fluence is up to 1.3 × 10^12^ n/cm^2^. Additionally, the leakage current variations and the threshold voltage offsets of the two types of devices have little difference between on-state and off-state cases during the neutron irradiation. Some previous studies showed that the vacancy defect and interstitial atom defect produced by the DDD effect would cause the leakage current and threshold voltage degradation in silicon devices. Moreover, the initial defect concentrations produced by the DDD effect are independent of the bias applied [12,13]. This may be the possible reason why the bias voltage of the two-type GaN HEMTs is not very sensitive to the DDD effect.

**Figure 7:**
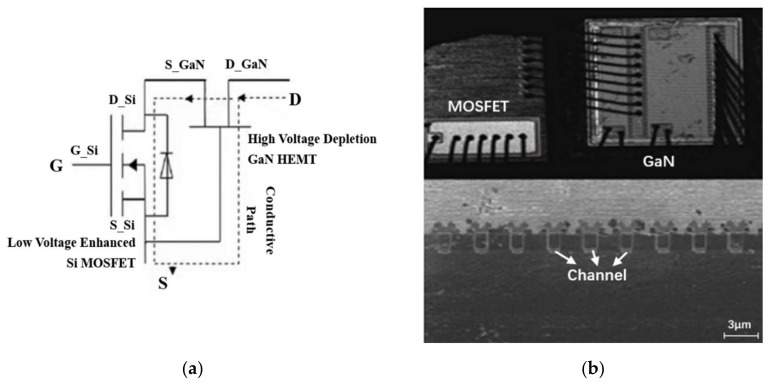
Schematic diagram of (**a**) the circuit structure of the GaN HEMT based on the cascode structure and (**b**) the internal MOSFET cross-section.

An analysis of data in Figure 5 shows that the drain current degradation of the GaN HEMT based on the cascode structure and the p-GaN gate exposed to both DDD and TID effect at accumulated gamma-ray doses of 800k rad(Si) exhibit obviously lower than the sum of the two separated effects. It indicates that the cascode structure and the p-GaN gate GaN HEMT are sensitive to the synergistic effect of DDD and TID. Further analysis of the internal circuit structure of GaN HEMT based on the cascode structure can be seen in Figure 7a. The circuit is composed of a low-voltage silicon metal–oxide–semiconductor field-effect transistor (MOSFET) and high voltage depletion-mode GaN HEMT. Figure 7b shows a cross-sectional view of the low-voltage silicon MOSFET extracted by the scanning electron microscope (SEM). It can be found that the low-voltage silicon MOSFET is an N-channel MOSFET with a trench structure. Under the off state, the drain-gate terminal of the depletion-mode GaN HEMT and the source-drain channel of silicon MOSFET is the main current-leakage paths. Previous reports [14,15,16] showed that N-channel MOSFET is very sensitive to TID and that the drain current increases with the accumulated gamma-ray dose. In addition, the defect caused by neutron irradiation in the silicon layer and Si-SiO_2_ interface could lead to an increase in the channel resistance and a decrease in the carriers’ mobility in the MOSFET device. Consequently, the leakage current of GaN decreases. This is a possible reason why the drain current of the GaN HEMT based on the cascode structure is obviously suppressed under the synergistic effect of the DDD and TID effect.

**Figure 8:**
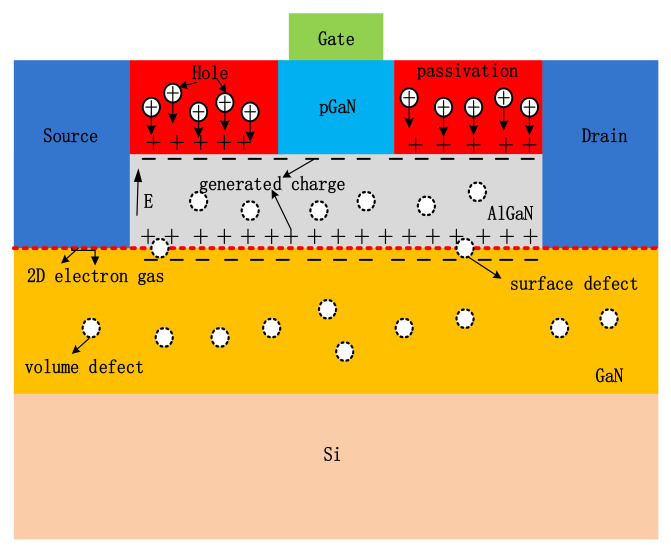
Schematic diagram of the electron-hole pairs transport process induced by the interaction of neutrons and gamma-rays in GaN HEMT.

The distribution of radiation-induced charges and defects in p-GaN gated GaN HEMT under the synergistic effect of DDD and TID effect is shown in Figure 8. During gamma-ray irradiation, electron-hole pairs are generated in the passivation layer of the device. Under the forward gate electric field, the generated holes drift towards the interface of the passivation layer and the AlGaN barrier layer. These holes could be captured by deep-level traps near the interface, forming the trapped charges. Additionally, this could lead to the form of the built-in electric field between the passivation layer and the channel region. The direction of the built-in electric field is opposite to that of the external gate electric field, which would reduce the depletion effect of the p-GaN layer on two-dimension electron gas (2DEG). Thus, the leakage current in the source-drain channel increases. Meanwhile, during the neutron irradiation, bulk defects and interface defects are possibly produced in the AlGaN barrier layer, the GaN buffer layer, the passivation layer, and the AlGaN/GaN interface. This will result in an increase in the channel resistance and the degradation of the 2DEG mobility, further leading to a leakage current decrease. This may be the main reason why the drain current of the p-GaN type GaN HEMT decreases significantly under the synergistic effect of the DDD and TID effect.

**Figure 9:**
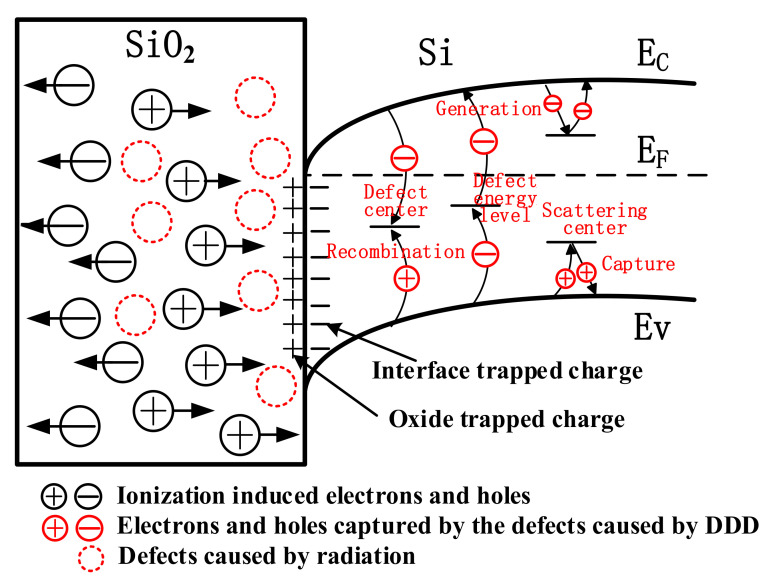
Distribution of charge in N-channel MOSFET induced by the synergistic effect of gamma-ray and neutron.

Analyzing the data in Figure 6, it is interesting that the negative shift of the threshold voltage of the GaN HEMT based on the cascode structure is −6.0 V under the combined effect of neutron and gamma-ray. This value is not equal to −4.6 V, which is the sum of the threshold voltage shift caused by the neutron and the gamma-ray irradiation. It confirms that GaN HEMT has an obvious synergistic effect on DDD and TID effects. Previous studies [17,18,19,20,21,22] have shown that neutron irradiation and gamma-ray irradiation could lead to a negative shift of the threshold voltage in the N-channel MOSFET and a positive voltage shift in the depletion mode GaN HEMT. Additionally, MOSFET is more sensitive to neutron and gamma-ray irradiation than the depletion mode GaN HEMT, leading to the negative shift of the threshold voltage of GaN HEMT based on the cascode structure under the synergistic effect of DDD and TID effect. Figure 9 shows the distribution of the radiation-generated charge in the N-channel MOSFET induced by the synergistic effect of the DDD and TID effects. Electron-hole pairs are generated in the oxide layer of the MOSFET under gamma-ray irradiation. Electrons rapidly move to the gate electrode and the holes quickly move to the SiO_2_/Si interface under the positive electric field at the gate port. During this processing, electrons are captured by the donor traps in the oxide layer, forming the oxide trap charges, and holes are captured by the acceptor traps in the SiO_2_/Si interface, forming the interface trap charges. The shift of the threshold voltage resulting from charge trapping in the MOSFET can be calculated according to (1).

(1) $$\Delta V_{th}=\frac{d_{ox}g\;q}{\varepsilon_{sio_{2}}}(\Delta N_{ot}-\Delta N_{it})$$

where$\;\varepsilon_{SiO_{2}}$ is the dielectric constant of *SiO_2_*, *q* is the elementary charge, *d_ox_* is the oxide layer thickness, $\Delta N_{ot}\;$is the number of the radiation-induced oxide trap charge and$\;\Delta N_{it}$ is the number of the radiation-induced interface charge. The negative *V_th_* shift is proportional to the net charge accumulation caused by radiation in the oxide layer, which is the main reason for the negative *V_th_* shift in the MOSFET. When exposed to neutron irradiation, different types of defects are produced in the oxide layer, increasing the defect density in the device [23,24]. These radiation-induced defects may capture the holes induced by gamma-ray irradiation and further increase the number of trap charges in the oxide layer. This may be responsible for the obvious increase in the threshold voltage negative shift under the synergistic effect of the DDD and TID effects. While the *V_th_* shift of the p-GaN type GaN HEMT is about −0.2 V under the synergistic effect of DDD and TID effects. The value is mostly the same as the sum of the threshold voltage shift caused by the single neutron and gamma-ray irradiation. When the GaN HEMT is exposed to neutron irradiation, donor traps are produced [25,26] in the p-GaN layer, which weakens the depletion effect of p-GaN on 2DEG, leading to a negative shift of the threshold voltage. For the gamma-ray irradiation, additional insulating layers and interface traps are produced in the insulating layer and the insulating layer/AlGaN interface. Because of the thin gate insulator layer and the low transfer rate of the interface trap charge, the number of the radiation-generated charges is greatly suppressed, which is hard to impact on the 2DEG in the channel, leading to the change of the threshold voltage [27,28]. Therefore, the negative *V_th_* shift of p-GaN GaN HEMT caused by the synergistic effect of DDD and TID effect is mainly affected by neutron irradiation.

## 5. Conclusions

This work investigated the synergistic effect of the DDD and TID effects for enhancement-mode GaN HEMT based on the p-GaN gate and the cascode structure using a 14 MeV neutron high voltage multiplier and ^60^Co gamma-ray irradiation. The influence of the DDD effect on the TID effect of GaN HEMT with different structures under the on-state and off-state were investigated. The experimental results show that the GaN HEMT based on the cascode structure is much more susceptive to the synergistic effect of the DDD and TID effects than the p-GaN gate GaN HEMT. Additionally, the failure mechanisms of the synergistic effect of the DDD and TID effects were proposed. The bulk and interface defects caused by neutron irradiation in the GaN HEMT will lead to an increase in channel resistance and the degradation of 2DEG mobility, which inhibits an increase in the drain current. This may be the main reason why the drain current of the GaN HEMT based on the p-GaN gate structure decreases significantly under the synergistic effect of the DDD and TID effects. In addition, Si MOSFET is a possible reason why the cascode structure GaN HEMT is sensitive to the synergistic effect of the DDD and TID effects. Defects in the oxide layer induced by neutron irradiation may capture the holes induced by gamma-ray irradiation, leading to a further increase in the number of oxide trap charges. This may be the reason for the significant increase in the threshold voltage shift of the cascode structure GaN HEMT.

## Figures and Tables

**Table 1:** Parameters of the device.

	Type	BV	Ron	Manufacturer
GS0650111L	P-type gate	650 V	150 mΩ	GaN Systems
TP90H180PS	Cascode	900 V	205 mΩ	Transphorm

## Data Availability

Data available in a publicly accessible repository.
